# Triphasic Development of the Genetic Code

**DOI:** 10.1021/acs.chemrev.3c00915

**Published:** 2024-08-01

**Authors:** Tze-Fei Wong

**Affiliations:** Division of Life Science and Applied Genomics Center, Hong Kong University of Science & Technology Hong Kong, China

## Abstract

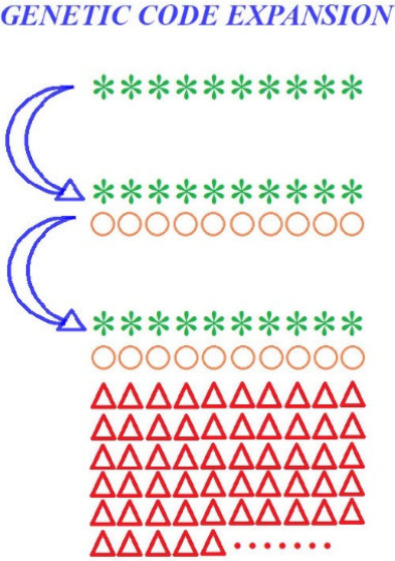

The genetic code contains an alphabet of genetically encoded amino acids. The ten Phase 1 amino acids, including Gly, Ala, Ser, Asp, Glu, Val, Leu, Ile, Pro and Thr, were available from the prebiotic environment, whereas the ten Phase 2 amino acids, including Phe, Tyr, Arg, His, Trp, Asn, Gln, Lys, Cys, and Met, became available only later from amino acid biosyntheses. In the archaeon *Methanopyrus kandleri*, the oldest organism known, the standard alphabet of 20 amino acids was “frozen” and no additional amino acid was encoded in the subsequent 3 Gyrs. Four decades ago, it was discovered that the code was frozen because all the organisms were so well adapted to the standard amino acids that oligogenic barriers, consisting of genes that are thoroughly dependent on the standard code, would cause loss of viability upon the deletion of any one amino acid from the code. Once the reason for the freezing of the code was ascertained, procedures were devised by scientists worldwide to enable the encoding of novel noncanonical amino acids (ncAAs). These encoded Phase 3 ncAAs now surpass the 20 canonical Phase 2 amino acids in the code.

## 1 Introduction

**Figure 1:**
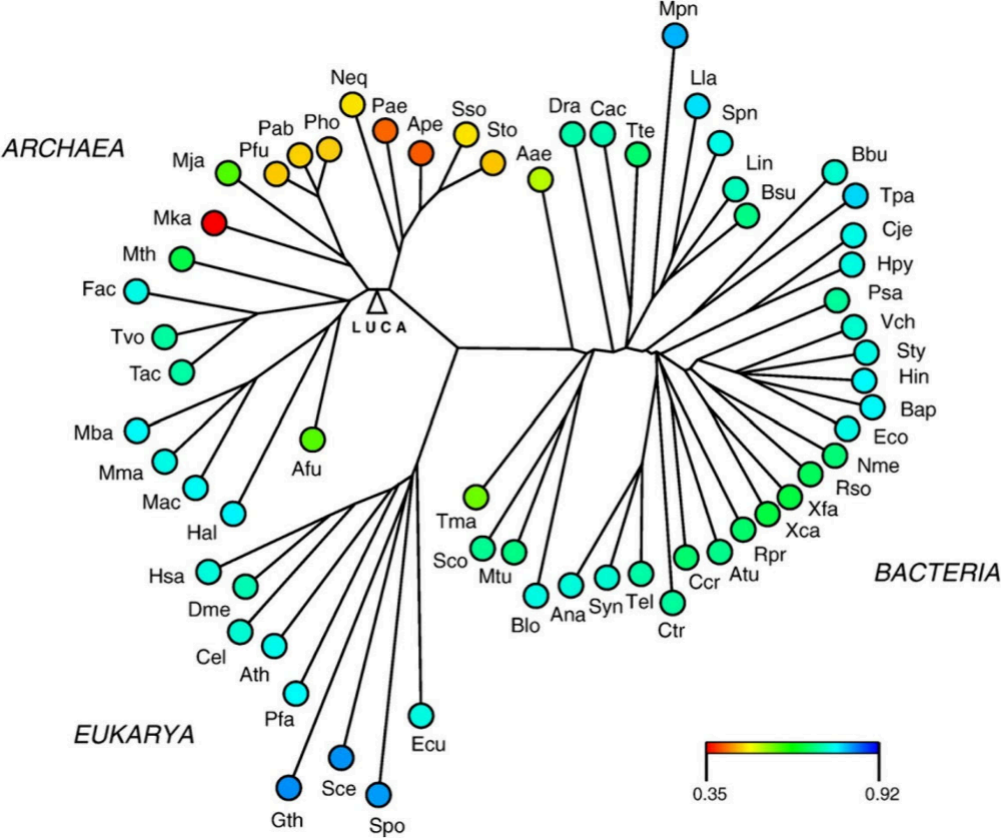
Evolution of interalloacceptor tRNA distances in the three biological domains. 0.35 on the distance scale indicates the closest distance between alloacceptor tRNAs. Reproduced with permission from ref (4). Copyright 2007 Elsevier.

Living matter is founded on collaboration between nucleic acids and proteins through the genetic code. The success of this collaboration has bestowed on Earth a surface layer consisting of both inorganic and organic chemicals, which is extremely favorable for the emergence of living systems. While it is not known how many nucleobases were required to encode an amino acid in the earliest versions of the code, evidence indicates that, by the time the precells gave rise to the Last Universal Common Ancestor (LUCA) in the phylogenetic neighborhood of the archaeon *Methanopyrus kandleri* (Mka) some 3 Gyrs ago,^1,2^ the genetic code was already encoding the 20 proteinogenic amino acids in the Standard Genetic Code (SGC) today using triplet nucleobase codons. Notably, attributes of the code besides its invariant 20-amino acid alphabet have continued to undergo evolutionary changes, exemplified by the wobble rules that assign two anticodons to amino acids endowed with 4-codon boxes in Mka, two to three anticodons in other archaea, and varied numbers of anticodons in bacteria and eukaryotes^3^ (Table 1). In parallel, the sequences of alloacceptor tRNAs that accept different amino acids are clustered close to one another in sequence space in the primitive archaea near Mka (colored red, Figure 1), but farther apart from one another in bacteria and eukaryotes (colored green-blue), indicating that the alloacceptor tRNAome of living species shared similar sequences at first, but become more dispersed from one another as they evolve.^4^

**Table 1:** Anticodons Employed by Different Organisms. Reproduced with Permission from Ref (3). Copyright 2016 MDPI

**Stage**	**Anticodon**	**3rd Codon Base Read**	**Main Users**
I	UNN	U, C, A, G	Pre-LUCA organisms
II	GNN	U, C	Primitive Archaea
	UNN	A, G	
III	GNN	U, C	Majority Archaea
	UNN	A, G	
	CNN	G	
IV	GNN	U, C	Bacteria, Eukarya
	UNN	A, G	
	CNN	G	
	INN	U, C, A	
V	UNN	U, C, A, G in 1aa boxes	Mitochondria, Chloroplasts, *Mycoplasma*, *Stretoococcus, Borrelia, Lactococcus*

Such findings amply attested to the ongoing evolution of various aspects of the code, unlike their common amino acid alphabet, which was apparently frozen in time since the appearance of Mka. This has posed a 3 Gy enigma regarding the code, viz. why has the amino acid alphabet stopped evolving between Mka and modern organisms, barring indefinitely the admission of any additional encoded amino acid into the code.

## 2 Formation of RNA Genes

Because RNAs can store genetic information and also function as ribozymes and aptamers, whereas proteins excel as catalysts and specific ligand binders but cannot store genetic information, it was proposed that an RNA World was established when long RNA polymers could be synthesized on complementary linear templates to form double helices,^5,6^ long before the present-day Protein World. Initially, the double helices in the RNA World comprised overwhelmingly nonfunctional RNA sequences^7,8^ that would not melt to enable continued replication. However, this roadblock to continual replication could be overcome in the rare event that one strand of the double helix was endowed with aptamer or ribozyme activity. Aptamers could be formed toward a wide range of target molecules, including metabolites and proteins. Importantly, they can bind their targets with *K*_d_ values in the low nanomolar to micromolar range, and discriminate between substrates as efficiently as monoclonal antibodies.^9^ Consequently, upon the exposure of a population of random RNA helices to a wide spectrum of aptamers and ribozymes, the latter would adhere to those helices containing an aptamer- or ribozyme-encoding strand, and loosen up the helix to permit replication of its strands through the mechanism *REplicator Induction by Metabolites* (REIM) for sustained replication of aptamers and ribozymes, while the random nonfunctional RNA helices remained unmelted, underwent decay and released building blocks to support the replication of the functional RNAs as primordial RNA genes,^10^

**Figure 2:**
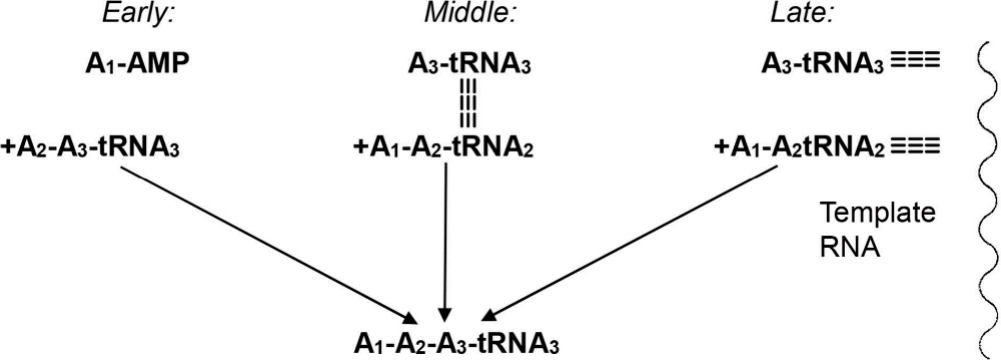
Successive stages in the evolution of the mechanism for the synthesis of a tripeptide-tRNA. Reproduced with permission from ref (14). Copyright 1991 Springer Nature.

With only the four side chains of U, C, A and G, the RNAs searched among the prebiotically available small molecules for suitable post-transcriptional modifiers to assist their ribozyme and aptamer activities. The N^6^-threonyl-carbamoyladenosine (t^6^A) modifier is present in the tRNA anticodon loop in all three biological domains, playing a crucial role in maintaining decoding accuracy during protein synthesis. The formations of t^6^A and N^6^-hydroxynorvalyl-carbamoyladenosine (hn^6^A), accomplished early from prebiotic Thr and hydroxynorvaline,^11^ were representative of the earliest interactions between amino acid and RNA. The N^5^-taurinomethyluridine (taum^5^U) modifier was synthesized from taurine imported into mitochondria from cytoplasm,^12^ whereas the synthesis of the Glu-Q modifier was catalyzed by a Glu-Q-tRNA(Asp) synthetase encoded by the *yadB* gene through glutamylation of a queuosine at the tRNA(Asp) anticodon wobble position.^13^ Such diverse occurrences of amino acid-nucleoside pairings were suggestive of their precursor roles in the utilization of aminoacyl-RNA and peptidyl-RNA as intermediates in ribosomal genetic encoding of peptide sequences^14^ (Figure 2).

## 3 Coevolution of Codons and Amino Acid Biosynthesis

**Figure 3:**
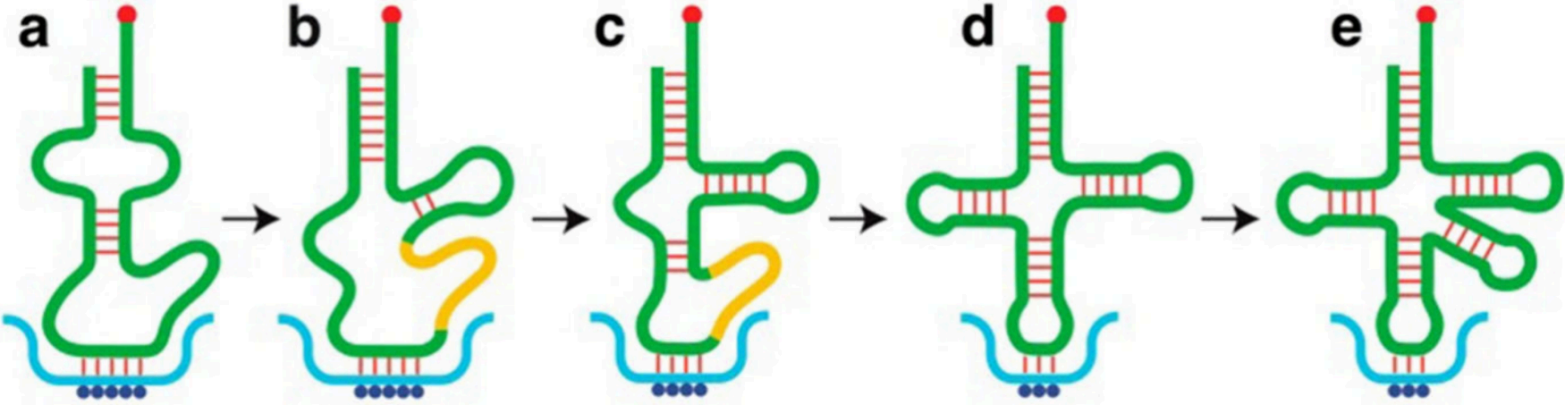
Plausible adaptation of a self-aaRS to function as a tRNA. Reproduced with permission from ref (3). Copyright 2016 MDPI.

In the transition from the RNA World to the Protein World, some of the ribozymes and RNA aptamers might turn into messenger and rRNAs. The ribozymal self-charging aminoacyl-RNA synthetases (or “self-aaRSs”) were found to be endowed with the twin ability to activate a specific amino acid, and to transfer the activated amino acid to a tRNA acceptor at the 3′–OH terminus.^15^ Such self-aaRSs would be exceptionally well suited to undergo adaptation as tRNAs for distributing the codons of a genetic code to different amino acids^3^ (Figure 3).

Numerous proteinogenic amino acids are produced from precursor amino acids through amino acid biosynthetic pathways. Analysis of the Standard Genetic Code revealed that the product amino acids are often encoded by codons situated next to the codons (viz., one nucleobase apart) of their precursors. A number of amino acids derived from Asp received third-row codons, and a number of amino acids derived from Glu received second-row codons, while the column-1 codons translated the three biosynthetically related Leu, Ile and Val. All three termination codons were located in row-1, which was consistent with the use of row-1 to store spare codons for additional Phase 2 amino acids. On this basis, the *coevolution theory* proposed that the code coevolved with amino acid biosynthetic pathways: the ten early arriving amino acids Gly, Ala, Ser, Asp, Glu,Val, Leu, Ile, Pro and Thr were available from the prebiotic environment and constituted the Phase 1 code, whereas the ten later-arriving amino acids Phe, Tyr, Arg, His, Trp, Asn, Gln, Lys, Cys and Met, which received encoding subsequently when they became abundant from biosynthesis, made up the Phase 2 code.^16,17^ Years later, this proposal was validated when the carbonaceous Antarctica meteorite CR2^18^ as well as high energy irradiation^19^ gave rise to the Phase 1 amino acids and no Phase 2 amino acid. That no codon from row-4 was assigned to any Phase 2 amino acid, and the vanishing concentrations of Gln, Asn and aromatic amino acids under prebiotic conditions due to their thermal or UV degradation,^20,21^ supported the dissimilar sources of the Phase 1 and Phase 2 amino acids.

The ribozymal “flexizymes” (Fxs) can bypass specific aaRS-tRNA pairings to yield peptide bonds, and their twin ability to carry an activated amino acid and transfer it to the 3′–OH terminus of a tRNA type acceptor resembles that of the self-aaRSs. Moreover, the newer Fxs are capable of cooperating with the ribosome and bacterial elongation factor EF-P to build peptide bonds. They are therefore versatile adaptations of self-aaRSs useful for introducing ncAAs into peptides in cell-free systems through amino acid activation by non-ATP activators.^22−25^ The elaboration of the Fxs from esterifying enzymes to ribosome-type systems for peptide synthesis confirms the plausibility of the self-aaRS origin of tRNA (Figure 3) as the starting point of ribosomal translation and genetic code.

Unexpectedly, the analysis of “urzymes”, i.e. the ancient forms of modern enzymes, enabled verification of the classification between Phase 1 and Phase 2 amino acids. Two highly conserved consensus sequence motifs, HIGH and KMSKS, in the Rossmann fold of various Class I aaRSs are implicated in the catalysis of both amino acid activation and acyl-transfer to tRNA. Using the AVGA and AMSAS forms of these two motifs in the urzyme of LeuRS of *Pyrococcus horikoshii*, replacing the usual Phase 2 amino acids histidine and lysine by Phase 1 alanine respectively resulted only in modest changes in enzyme activity, which proved the absence of Phase 2 histidine and lysine at the urzyme stage of evolution, and therefore the coevolution theory.^26^ Furthermore, the high catalytic capability of a number of aaRS urzymes suggests that there could be a substantial transitional period between the RNA World and Protein World, during which Phase 2 amino acids were added to the code.^27^

Multiple mechanisms likely influenced the placement of the 20 encoded amino acids in the genetic code. Direct interaction between RNA and amino acids through both the alpha-carbon and the amino acid side chain yielded *K*_D_ of 10^–4^ to 10^–6^ M for the favorable amino acids,^28^ and the prominent role played by an Alanine World in the formation of prebiotic alpha helices would favor early usages of GC codons followed by GCU and GCUA.^29^ The lack of any Phase 2 amino acid with a row-4 codon was consistent with the use of row-4 codons only to encode Phase 1 amino acids in the earliest codes. Later, when Phase 2 amino acids entered into the code, row-3 codons were enriched with amino acids of the Asp biosynthetic family, while row-2 codons were enriched with amino acids of the Glu biosynthetic family. The row-1 codons, which included all three termination signals, likely provided storage for spare codons required by the Phase 1-to-Phase 2 code expansion.

## 4 Oligogenic Barriers Froze the Genetic Code

**Figure 4:**
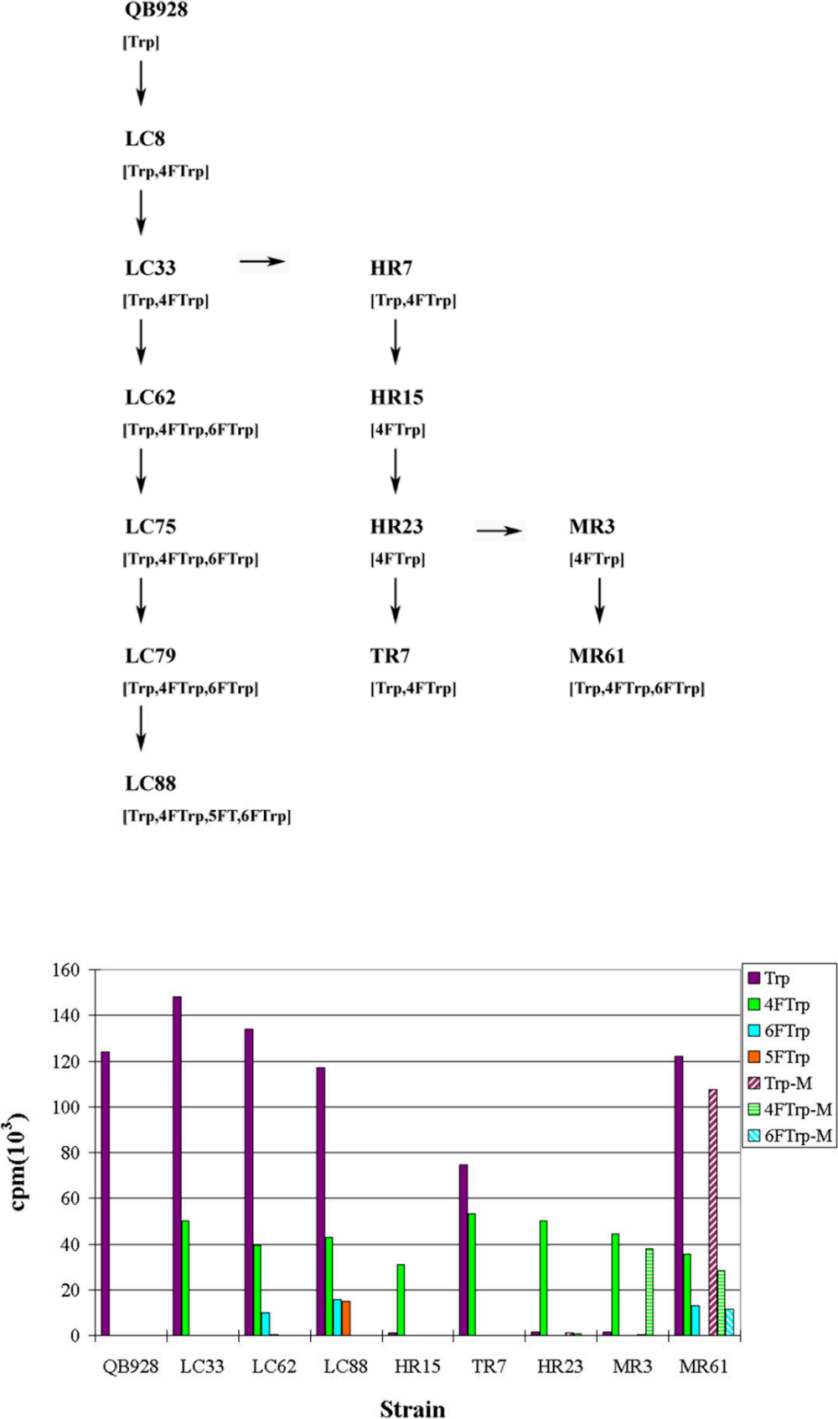
Isolation of *B. subtilis* QB928-derivative strains (top); and their growth rates on different Trp analogues (bottom). Reproduced with permission from ref (31). Copyright 2010 PLoS.

**Figure 5:**
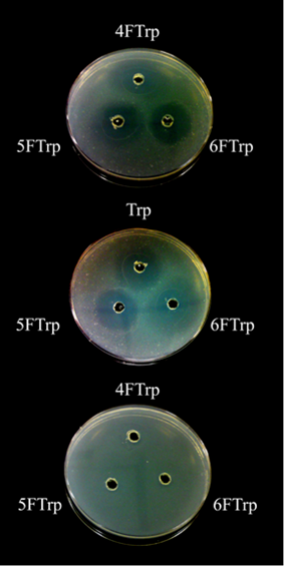
Inhibition by Trp or different Trp analogues indicated by cleared zones around wells containing them on a lawn of QB928 cells (top), on a lawn of HR 23 cells (middle), or on a lawn of LC88 cells (bottom). Reproduced with permission from ref (31). Copyright 2010 PLoS.

Since an impressive array of polypeptides related to the translation process, including initiation factors, elongation factors, ribosomal proteins, and aaRSs, have varied between species from different biological domains, it was not reasonable to suppose that the evolution of the Phase 2 code would cease without cause for 3 Gyrs. Accordingly, to better understand the situation, we sought to restart code evolution by attempting to displace encoded Trp by its 4-fluoroTrp (4FTrp) analogue through plating the cells of Trp synthesis-deleted *Bacillus subtilis QB928* repeatedly on agar containing high 4FTrp and minimal Trp. This led to the isolation of strain LC8 that showed a low level of growth on 4FTrp, then strain LC33 which grew on 4FTrp at 30% the rate on Trp, and eventually strains HR15 and HR23 which grew well on 4FTrp but not on Trp. Moreover, Trp acted like a typical inhibitory analogue against the growth of HR23 on a 4FTrp medium. HR23 could revert to strain TR7 that grew once more on Trp or 4FTrp, or to strain MR61 that grew well not only on Trp or 4FTrp, but also more slowly on 6FTrp. The results obtained with MR61 were little affected by the withdrawal of Met supplement from the growth media, showing that the reverted growth on Trp was not merely a turnover of the N-terminal fMet residue. In addition, the LC-series of mutants yielded strain LC88, which grew on Trp, 4FTrp, 6FTrp as well as 5FTrp (Figure 4).^30,31^Figure 5 demonstrates the patterns of mutual inhibitions of cell growth between these different indole-amino acids: Top: each of 4FTrp, 5FTrp and 6FTrp placed in a hole brought about a clear zone on a lawn of wildtype QB928 cells grown on Trp, indicative of their respective inhibitions of QB928-growth; middle: each of Trp, 5FTrp and 6FTrp brought about a clear zone on a lawn of HR23 cells grown on 4FTrp, indicative of their respective inhibitions of HR23-growth; bottom: none of 4FTrp, 5FTrp and 6FTrp brought about a clear zone on a lawn of LC88 cells grown on Trp because of the ability of LC88 to grow on Trp, 4FTrp, 5FTrp or 6FTrp.

DNA sequencing of the genomes of wildtype QB928 and a number of its derivative strains showed that it required 12 missense, nonsense, and frameshift mutations in 11 genes to convert QB928 to LC33 to allow growth on 4FTrp, but as many as 34 missense, nonsense, and frameshift mutations to convert LC33 further to HR23 to disallow growth on Trp. In contrast, two different mutations of HR23 sufficed to reverse its loss of growth on Trp in the revertants TR7-1 and TR7-2; both of these mutations were located at the outer clawlike region of RNAP.^32^ A comparable situation might have prevailed during the formation of the Phase 2 code, when the cell populations were modest in size, and the code was mutating too frequently for the buildup of strongly restrictive oligogenic barriers.

The findings on the *B. subtilis* QB928-derivative strains showed that 12 Trp residues in the proteome fit their microenvironments so uniquely that their replacement by 4FTrp was nonviable to the cells unless the microenvironment was mutated in each instance to render the replacement of Trp by 4FTrp acceptable for the cells; these 12 genes thus constituted an oligogenic barrier against the usage of 4FTrp. On the other hand, the microenvironments of another 34 genes in the LC33 strain had to be mutated to abolish the viability of Trp-grown cells. Consequently, these experiments confirmed that the encoded amino acid alphabet has been mutable intrinsically throughout the entire history of life; and only strong oligogenic barriers had effectively blocked the deletion of any of the 20 encoded amino acids from the alphabet. Once these barriers were recognized, the 3 Gyr long pause in the evolution of the alphabet was terminated, and the Phase 3 genetic codes began.

## 5 Thawing of the Frozen Code

Although the Phase 3 codes started only four decades ago, three major approaches already have been developed for thawing the “frozen” genetic code and introducing ncAAs into proteins. As a result of these developments, the Phase 3 codes today can encode far more ncAAs than the 20 canonical amino acids (cAAs) in Phase 2 code.

### 5.1 Selective Pressure Incorporation

The application of repetitive mutations to unblock the usage of 4FTrp as effective growth substrate for various *B. subtilis QB928* derivative strains has been extended to *Esherichia coli,*(33) and selected strains of these cells can convert 4F-indole metabolically into 4FTrp followed by its direct incorporation into cellular proteins.^34^ Therefore, this ncAA has become in effect the modern version of a Phase 2 amino acid, being recruited straight into the code as it comes off the biosynthetic pipeline.

### 5.2 Usages of Orthogonal Translation Components

Ribosomal protein synthesis typically depends on specific pairs of cognate translational or transcriptional components. Analysis by us revealed that when an *E. coli* aaRS preparation was employed to acylate tRNAs from a range of bacteria, the archaeon *Halobacterium cutirubrum*, and eukaryotic rat liver, wheat germ and yeast, high yields of acylation were obtained with numerous bacterial tRNAs, whereas low yields were obtained with a majority of archaeal and eukaryotic tRNAs (Table 2); this was also the case when the bacterial *R. spheroides* aaRS preparation was employed. In contrast, a yeast aaRS preparation readily yielded high levels of acylation of yeast or wheat germ tRNAs.^35^ These findings pointed to the existence of a strong preference of bacterial aaRS for reaction with bacterial tRNAs instead of archaeal and eukaryotic tRNAs.

**Table 2:** Yields of aa-tRNA Formation by *E. coli* Aminoacyl-tRNA Synthetases Acting on tRNAs from Different Species. Reproduced with Permission from Ref (35). Copyright 1980 NRC Research Press

**tRNA**	**Phe**	**Leu**	**Asp**	**Lys**	**Arg**	**Tyr**	**Met**	**Val**	**Ser**	**Thr**	**His**	**Pro**
*Agrobacterium tumefaciens*	149	118	77	12	67	93	108	67	70	109	45	101
*Arthrobacter luteus*	47	38	2	27	58	19	61	32	27	63	15	89
*Bacillus stearothermophilus*	164	180	58	106	114	128	115	131	64	140	44	110
*Bacillus subtilis*	156	208	156	71	119	187	137	87	91	97	76	94
*Escherichia coli*	100	100	100	100	100	100	100	100	100	100	100	100
*Halobacterium cutirubrum*	2	0.4	3	2	0.4	0.5	29	30	0	69	7	1
*Micrococcus luteus*	20	48	2	18	88	34	19	14	65	67	41	88
*Myxococcus xanthus*	176	159	114	11	78	104	99	61	72	105	29	99
*Rhodopseudomonas sphaeroides*	54	100	12	75	58	4	61	44	63	108	6	86
*Thermus aquaticus*	51	80	7	28	72	2	65	24	102	91	41	87
Rat liver	2	0.4	0.5	1	16	0.3	6	3	0	2	7	2
Wheat germ	8	2	2	5	62	3	25	2	1	56	24	5
Yeast	1	2	1	14	0.5	4	43	1	4	69	10	15

The domain-dependent aaRS-tRNA reactivity has been utilized in the invention of specially designed aaRS-tRNA pairs to introduce ncAAs into proteins. By charging a yeast suppressor tRNA(Phe) with the ncAA *p*-fluoroPhe, the charged *p*-fluoroPhe-tRNA could function orthogonally, i.e. independently, from the *E. coli* host’s own tRNA-aaRS pairs and incorporate *p*-fluoroPhe into proteins.^36^

The utilization of orthogonal components to incorporate ncAAs into proteins has been applied to different translational components including aaRSs, tRNAs, mRNAs, translational factors and ribosomes for the purpose of enlarging the variety of encoded ncAAs.^37^

### 5.3 Use of Extra DNA Basepairs

**Figure 6:**
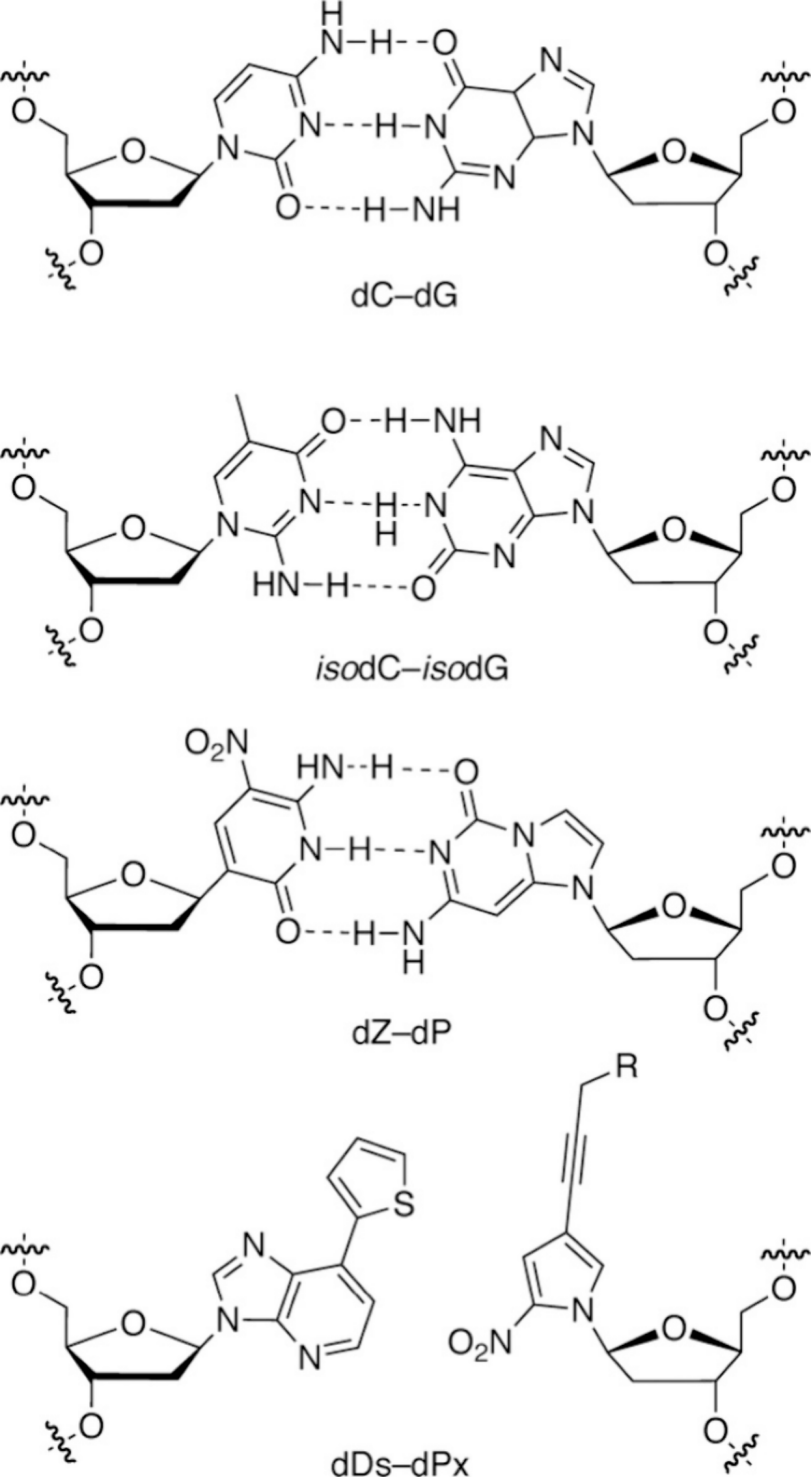
A canonical (top) and three noncanonical (other three) basepairs in DNA. Reproduced with permission from ref (37). Copyright 2021 ACS.

Canonical genes consist of four kinds of deoxyribonucleotides and accommodate 64 triplet codons. Use of three or four pairs of complementary deoxyribonucleotides enhances the number of available mRNA triplet codons, making possible the encoding of a greater number of amino acids within the same codon table.^38^Figure 6 illustrates the canonical deoxy C-G basepair and three noncanonical basepairs currently under active investigation.^38^ Notably, there is no hydrogen bond between the dDs-dPx basepair, overturning the long-held view that hydrogen bonds are essential for complementary basepairing in nucleic acids.

## 6 Discussion

The acceptance of ncAAs for genetic encoding generates a panorama of Phase 3 codes devised by scientists worldwide to explore a hitherto totally unknown Protein World with a flexible alphabet. A range of technologies have been developed, and new aspects of medicine, health, and industry have been opened to pursuit. The results obtained revealed the immense potential of *genetic code expansion* in terms of the encoding of ncAAs and their evident utility in medicine and food production.^37,39−41^ However, the rapid acquisition of encoded ncAAs has not been matched by an equally rapid advance in their usage. Therefore, the foremost challenge in this field will be the intensified exploration of novel applications of the wide-ranging ncAAs encoded by the Phase 3 codes.

For example, it was proposed that the synthesis of covalent protein–lipid conjugates could open up unknown biochemistries.^42^ Since cardiovascular diseases often stem from atherosclerosis and calcification on the inner lining of blood vessels, using ncAAs with monosaturated fat or other lipoidal side chains to coat the inner wall of blood vessels might reduce the occurrence of thrombosis.

Cancers, like heart disease, are becoming increasingly frequent in the fast-growing world of the aged. The major determinants of cancer include oncogenes, cancer suppressor genes, F-box proteins for degrading oncogene products, and cancer driver genes. Machine learning has been emploved to identify recurrent germline copy-number-variations (CNVs) associated with cancer in the Caucasian and Korean populations.^43^ Somatic mutations of oncogenes and cancer suppressor genes play important roles in cancers, and there could be more than 200 cancer driver genes, the somatic mutations of which occur in a variety of cancers.^44^ In a study of 44 solid tumors, evidence suggests that new driver mutations included CNVs, indels, and single nucleotide variations (SNVs), and evidence indicated that a small number of mutations sufficed to confer a neoplastic phenotype. These findings underlined the important role driver-gene mutations play in cancer mortality, yet treatment of driver gene mutants with monoclonal antibody was limited in efficacy.^45^ In cases where monoclonal antibodies were ineffective against such mutations, ncAAs may be employed to enhance the affinity of the paratope on the antibody toward the epitope on the antigen; incorporate an alkylating side chain into the paratope to construct a covalent paratope-epitope linkage; or reduce the bulky antibody structure to a stabilized loop containing the paratope in order to expedite tighter paratope-epitope interaction.

In conclusion, the genetic encoding of amino acid sequences in proteins began in the RNA World with prebiotically available amino acids in Phase 1 codes. Biosynthesis brought the formation of numerous new amino acids, some of which were selected for encoding by the Phase 2 code, which has remained invariant for 3 billion years. Since code expansion was resumed through experimental intervention in 1983, a broad spectrum of ncAAs have received encoding by mRNA to form the still growing Phase 3 amino acid alphabet. That the flexible Phase 3 cAA+ncAA codes have been incorporated into both prokaryotic and eukaryotic proteomes is indicative that the Phase 3 codes will spread to some of the organisms on the Tree of Life, radically altering the nature of the living world.
